# Hemoglobin as a probe for estimation of nitric oxide emission from plant tissues

**DOI:** 10.1186/s13007-019-0425-9

**Published:** 2019-04-23

**Authors:** Neha Singh, Satish C. Bhatla

**Affiliations:** Laboratory of Plant Physiology and Biochemistry, Department of Botany, University of Delhi, Delhi, 110007 India

**Keywords:** Hemoglobin, Nitric oxide, NO scavenger, Methemoglobin, MnIP-Cu

## Abstract

**Background:**

Plant roots contribute significant amount of nitric oxide (NO) in the rhizosphere as a component of NO in the ecosystem. Various pharmacological investigations on NO research in plants seek to quench endogenous NO by using externally applied NO quenchers, mainly 2-phenyl-4,4,5,5,-tetramethylimidazoline-1-oxyl 3-oxide (PTIO) and its more soluble form-carboxy-PTIO (cPTIO). Owing to serious limitations in its application cPTIO is no more a desired compound for such applications.

**Result:**

Present work highlights the significance of using hemoglobin in the bathing solution to not only release endogenous NO from plant tissue but also to quench it in a concentration-dependent manner.

**Conclusion:**

The protocol further demonstrates the diffusibility of NO from intracellular locations in presence of externally provided hemoglobin. The proposed method can have widespread applications as a substitute to debatable and currently used cPTIO as a NO scavenger.

## Background

Plants constitute an important source of biological NO emission in the terrestrial ecosystem [1, 2]. Following the first observation of NO emission from herbicide-treated soybean leaves into the atmosphere [3], several studies have focused on NO emissions from detached plant tissues, cell suspensions and mitochondria [2, 4–7].

NO is a gaseous, lipophilic biomolecule which acts as a free radical with ability to diffuse across cell membranes, through the cytoplasm and migrate intracellularly as well as from cell to cell across the apoplast. It diffuses at a rate of 50 μm s^−1^. Its solubility is 1.9 mM in aqueous solutions at 1 atm pressure. Half-life of NO in biological systems is reasonably short, less that 10 s. The rapid movement and removal of cellular NO, makes it an ideal signaling molecule for cell to cell communication in plant tissues both in normal growth conditions and under stress [3, 8–12]. It is a versatile molecule that can migrate and act concurrently in different cellular compartments and in opposite directions. NO is biosynthesized in plants via multiple routes which are broadly classified as reductive and oxidative pathways. It is produced through both enzymatically in plastids, mitochondria, chloroplasts, and non-enzymatically in the apoplast [6, 13–19].

Pharmacological investigations on the modulation of plant growth and development by NO routinely employ PTIO and its more soluble form-cPTIO as a means to quench tissue NO. Of late, it has been reported that cPTIO usage as a NO scavenger exhibits duality in its action [20]. Depending on concentration, cPTIO can, at times, even contribute to further NO production, rather than serving as a NO quencher. cPTIO oxidizes NO by forming ^·^NO_2_ radical (NO + cPTIO → ^·^NO_2_ + cPTI), which in turn can react with NO to form N_2_O_3_ (^·^NO_2_ + NO → N_2_O_3_). Thus, a reliable substitute NO quencher is required for various applications. Our recently published observations provided some evidence for the probable role of hemoglobin added in the growth medium on its ability to quench endogenous NO in sunflower seedlings [21, 22].

Ubiquitous occurrence of non-symbiotic hemoglobin (Hb) suggests that it serves important functions in the regulation of plant metabolism [23–31]. Endogenous hemoglobin primarily transports oxygen to various regions. Hbs reversibly bind with oxygen and their rates of binding and dissociation differ depending on the type of Hb. It also binds and scavenges NO and regulates its bioavailability in the tissues. In *Arabidopsis thaliana,* non-symbiotic class 1 and 2 Hbs reduce nitrite to NO, and this reaction rate increase linearly with [H^+^] increasing [32]. NO thus produced exhibits a strong affinity for the ferrous heme, leading to the formation of iron-nitrosyl-heme complex (Fe(II)-NO) as the final reaction product [33]. Hemoglobin scavenges NO through dioxygenation reaction where NO reacts with oxygenated hemoglobin (OxyHb; HbO_2_) to produce methemoglobin (MetHb; in which heme iron is in ferric state) and nitrate. This reaction occurs at the rate of 6–8 × 10^7^ M^−1^ s^−1^ [34].

$${\text{HbFe(II)O}}_{ 2} + {\text{NO}} \to {\text{MetHb}} + {\text{NO}}_{3}^{ - }$$

Deoxygenated hemoglobin i.e. hemoglobin with ferrous heme iron, can also bind NO [35, 36]. Under these conditions, NO is no longer available for physiological functions in the tissues.

$${\text{HbFe(II)}} + {\text{NO}}\underset{\text{Slow}}{\overset{\text{Fast}}{\rightleftarrows}}{\text{HbFe(II)NO}}$$

These observations form the basis of current investigations to demonstrate the application of hemoglobin in the bathing medium as an effective scavenger of NO released from live plant tissue. The evidence from the present work demonstrates the ability of hemoglobin to scavenge NO from all cellular and apoplastic components of the tissue system. The methodology thus proposed offers an alternative approach to scavenge endogenous NO in various pharmacological studies in plants.

## Materials and methods

### Plant growth conditions

Sunflower seeds (*Helianthus annuus* L., var. KBSH 54) were washed with a liquid detergent (teepol) under running tap water, disinfected using 0.005% mercuric chloride and again washed under running tap water for 1 h. Seeds were then imbibed in distilled water for 2 h and placed on moist germination sheets irrigated with half-strength Hoagland nutrient solution. Seedlings were grown up to 2 days in dark at 25 °C. Sunflower seedlings showing uniform growth pattern were selected for various analyses.

### Analysis of relative NO quenching ability of hemoglobin

Concentrated stock solution of hemoglobin (Sigma-aldrich, USA) was prepared fresh in distilled water for immediate use. To estimate the NO quenching ability of exogenously applied hemoglobin, 2 d old seedling roots were incubated for 30 min in dark in the absence or presence of variable concentrations of hemoglobin ranging from 250 μM to 3 mM. Each tube contained three seedlings. NO released from seedling root in the bathing solution was analyzed using MnIP-Cu (a copper derivative of 4-methoxy-2-(1*H*-naphtho (2,3-d) imidazol-2-yl) phenol; MNIP-Cu [37]. Seedling roots dipped only in distilled water were served as control. Following incubation, the bathing solution from each tube was taken for estimation of NO released in solution by treating with 2.5 µM of MnIP-Cu. NO released was monitored spectrofluorometrically (ex. 385 nm, em. 492 nm) and relative change in fluorescence was plotted to evaluate relative extent of NO released from tissue and quenched by variable concentrations of hemoglobin in solution.

### Estimation of methemoglobin formation

Methemoglobin formation as a result of reaction between NO released from seedling roots and hemoglobin present in the solution was monitored spectrophotometrically at 406 nM. Oxygenated hemoglobin absorbs at a wavelength of 415 nM and its reaction with NO lead to the formation of methemoglobin which shifts the absorbance of the product (metHb) to 406 nM.

### Visualization of NO in seedling roots in the absence or presence of hemoglobin

To further validate the scavenging ability of exogenously provided hemoglobin, seedling roots were dipped in distilled water containing variable concentrations of hemoglobin (2–3 mM) for 30 min in dark. Seedling roots dipped in distilled water served as control. After incubation, seedling roots were then incubated in 50 µM of MnIP-Cu for 45 min (ex. 385 nm, em. 492 nm). Using confocal laser scanning microscopy (CLSM; Leica, Germany), root tips were visualized for NO localization both in the absence or presence of hemoglobin.

### Detection of nuclei in seedling roots using CLSM

Seedling roots were incubated with 4,6-diamidino-2-phenylindole (DAPI; 2 µg ml^−1^ in distilled water) for 2 min to localize nuclei using CLSM (ex. 360 nm and em. 460 nm).

### Co-localization of NO and mitochondria in the seedling roots

Seedling roots were incubated in 50 µM of MnIP-Cu solution for 45 min and then dipped in 300 nM of MitoTracker (Molecular Probes, USA) for 45 min. NO and mitochondria signals were co-localized in the root-tip tissues by CLSM at ex. 385 nm; em. 492 nm for NO and at ex. 554 nm; em. 576 nm for mitochondria. Co-localization rate and mean intensity of co-localization of NO and mitochondrial signal were calculated using software LAS-AF, version 2.7-9723.3.

### Statistical analysis

All experiments were performed at least thrice and statistically analyzed by SPSS 22.0 statistical program (SPSS Inc, Chicago, IL, U.S.A.) using One-Way ANOVA.

## Results

**Fig. 1:**
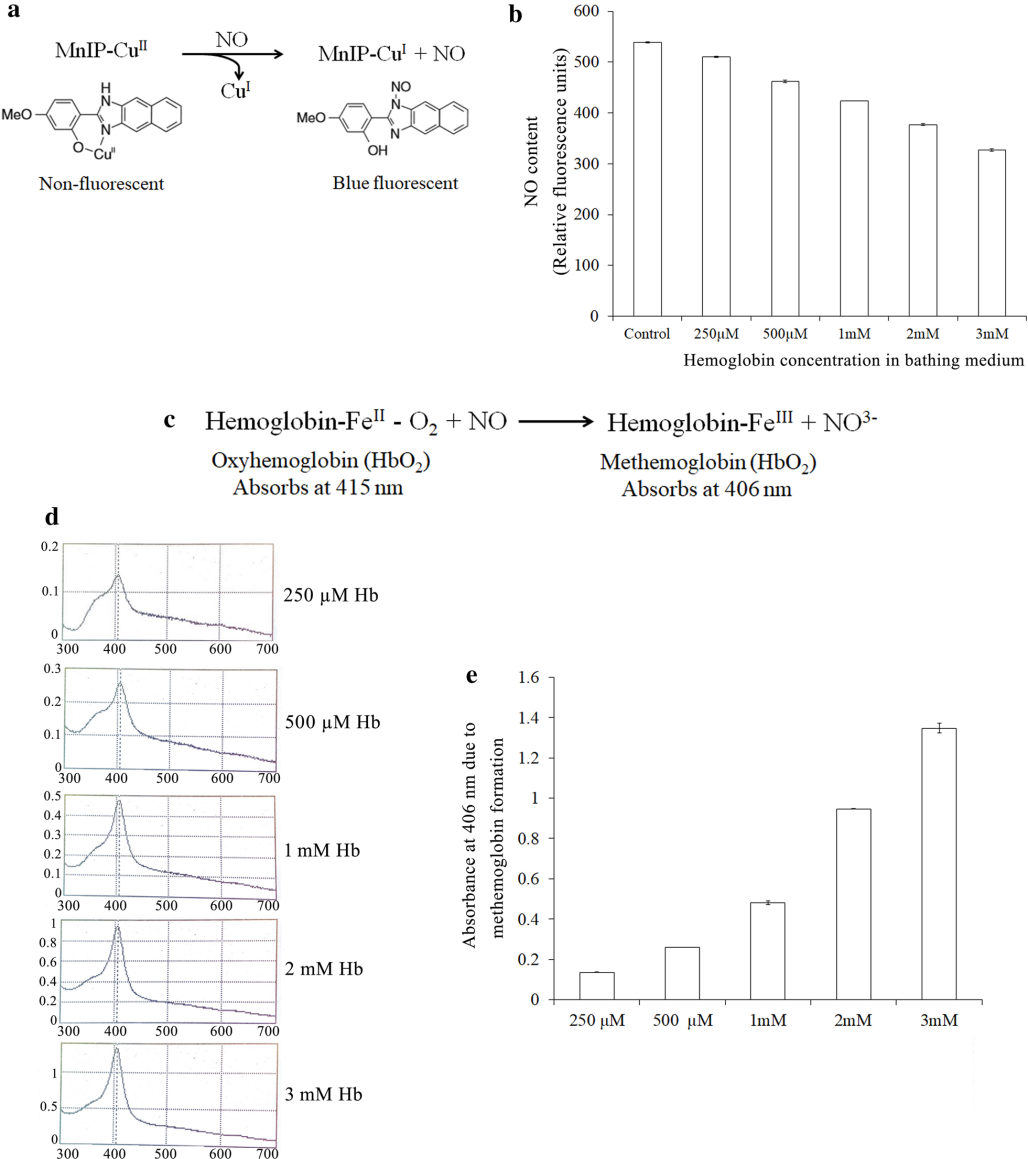
**a** Mechanism of action of MnIP-Cu, a novel probe for detection of NO in plant systems. **b** Quenching of NO released from seedling roots by exogenous hemoglobin (Hb) in a concentration (Hb)-dependent manner. NO released in solution from seedling roots bathed in varied concentrations of Hb was estimated using NO specific probe MnIP-Cu ex. 330 nm, em. 460 nm. **c** Conversion of oxyhemoglobin (HbO_2_) to methemoglobin in the presence of NO. **d** Absorbance peak for formation of methemoglobin in the presence of NO released from seedling roots in the solution bathed in varying concentrations of hemoglobin (250 µM to 3 mM) separately. **e** Increase in methemoglobin formation as a consequence of NO release from seedling roots in the bathing solution containing variable concentrations of hemoglobin (Hb). *Note*: Lower dosage of Hb in solution is insufficient in quenching NO from seedling roots

A novel fluorescence probe (a copper derivative of 4-methoxy-2-(1*H*-naphtho (2,3-d) imidazol-2-yl) phenol; MNIP-Cu; Fig. 1a) developed in the author’s laboratory in recent past for spectrofluorometric quantification and visualization of NO in live cells [37], has been used in the present work to examine the NO quenching ability of hemoglobin provided in the bathing medium. NO released from 2 d old, dark-grown sunflower seedling roots were monitored in the absence or presence of variable concentrations of hemoglobin (250 µM–3 mM) in the bathing solution. Since hemoglobin in solution binds with NO released from roots, resulting in methemoglobin (Hb-Fe^III^) formation and conversion of NO to NO^3−^, a Hb concentration-dependent decrease in the availability of free NO in solution is evident (Fig. 1b, c). Three millimoles of Hb leads to quenching of as much as 40% of NO released from roots as compared to control, thereby demonstrating the ability of externally available Hb to serve as a quencher of NO released from the tissue.

**Fig. 2:**
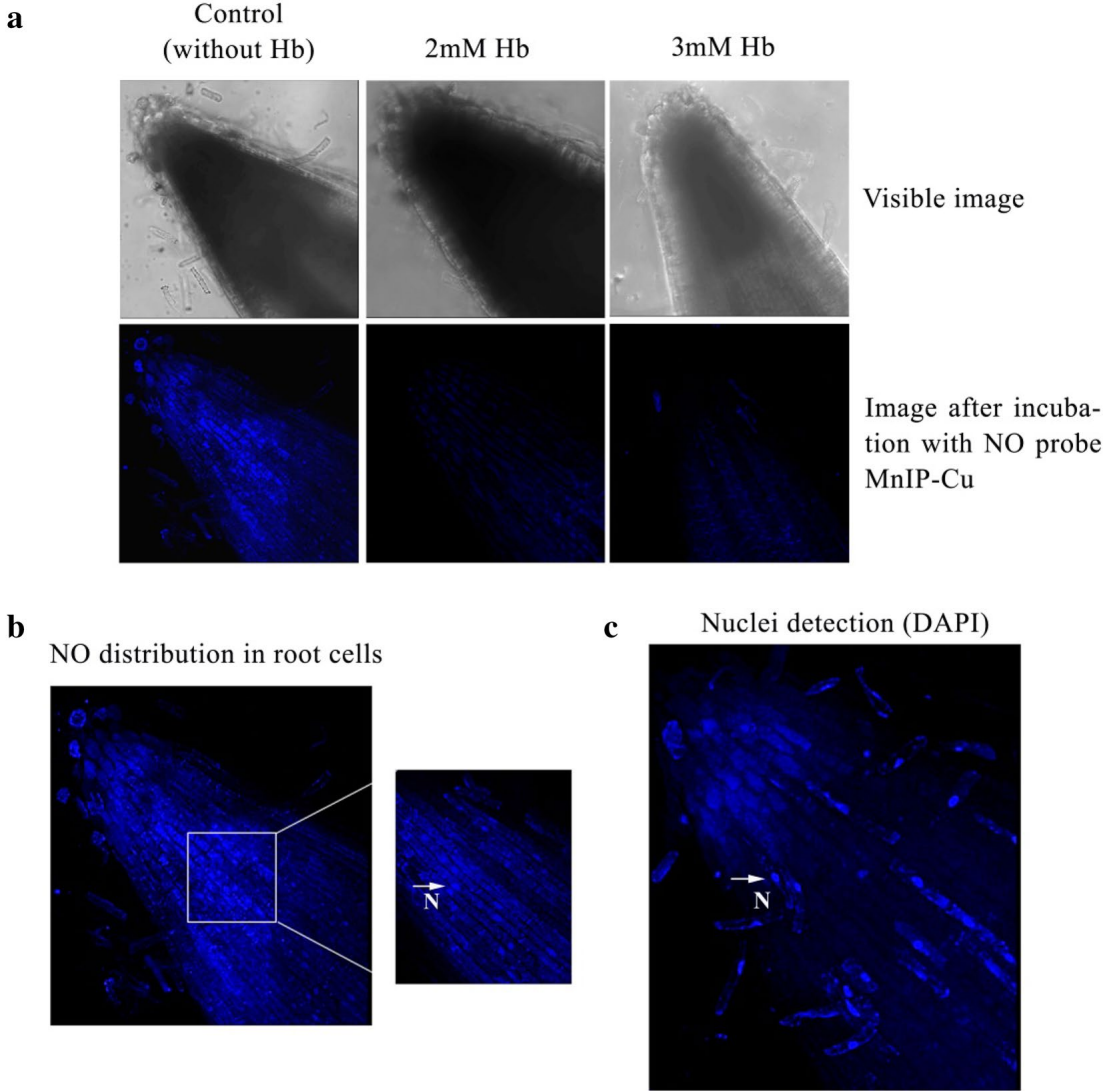
**a** Extracellular hemoglobin (Hb) as a quencher of endogenous NO from sunflower seedling roots. Seedling roots incubated in hemoglobin solution (2 mM and 3 mM) for 1 h followed by incubation in NO specific probe MnIP-Cu for 1 h. CLSM visualization of NO signal showed Hb concentration-dependent quenching of tissue NO. Roots were obtained from 2 day old dark-grown sunflower seedlings. Incubation medium without hemoglobin acts as control. **b** NO localization using CLSM in nuclei of root cells. Roots were obtained from 2 day old dark-grown sunflower seedlings. NO signal was visualized using MnIP-Cu. **c** Nuclei were visualized using DAPI. *N* Nuclei

**Fig. 3:**
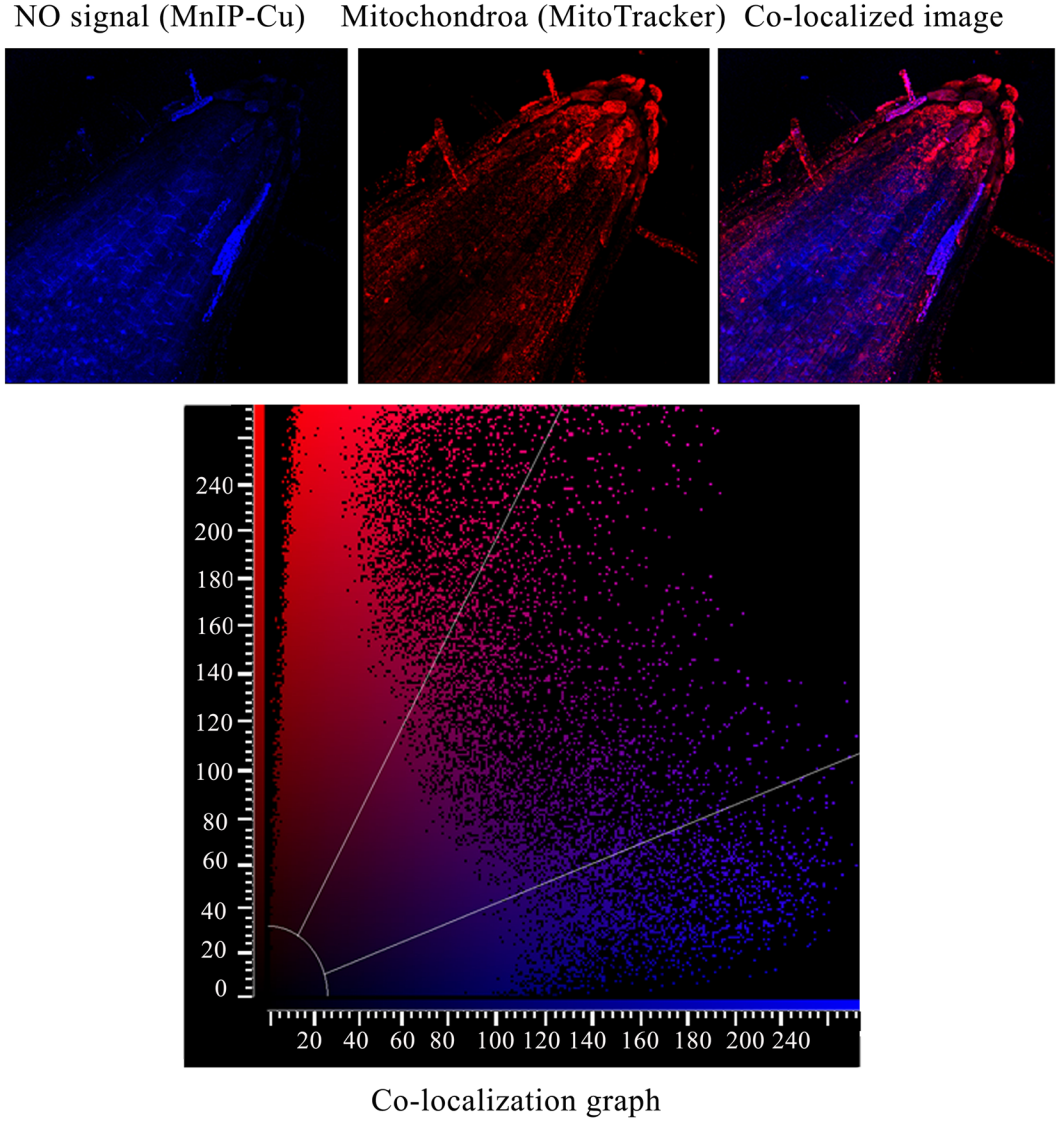
Co-localization of NO and mitochondria in 2 day old, dark-grown sunflower seedling roots. Data analysis was done using software LAS-AF, version 2.7-9723.3. Co-localization rate—51.44%. Mean intensity of co-localization of NO signal—41.41. Mean intensity of co-localization of Mitochondrial signal—37.39

Exogenous Hb (λ_max_ 415 nM) per se does not cross cell membranes (being a high molecular mass molecule of 64.5 kDa) but it can easily bind diffusible endogenous NO in a concentration (250 µM to 3 mM)-dependent manner and make it inaccessible as a free molecule in the bathing medium (forming methemoglobin; λ_max_ 406 nM) (Fig. 1d, e). This observation on Hb as a NO quencher carries significance for pharmacological investigations in plant cells where, so far, cPTIO have been extensively used as NO quenchers. Figures 2 and 3 provide detailed evidence for quenching of endogenous NO from sunflower seedlings root cells in response to externally provide Hb (2–3 mM). In addition to cytoplasm and apoplast, NO has also been localized in nuclei and mitochondria. The ability of exogenous Hb to trigger migration of NO from all these intracellular locations thus proves its (Hbs) scavenging ability (for NO) from all intracellular locations of the plant cells/tissues exposed to various pharmacological investigations.

## Discussion

**Fig. 4:**
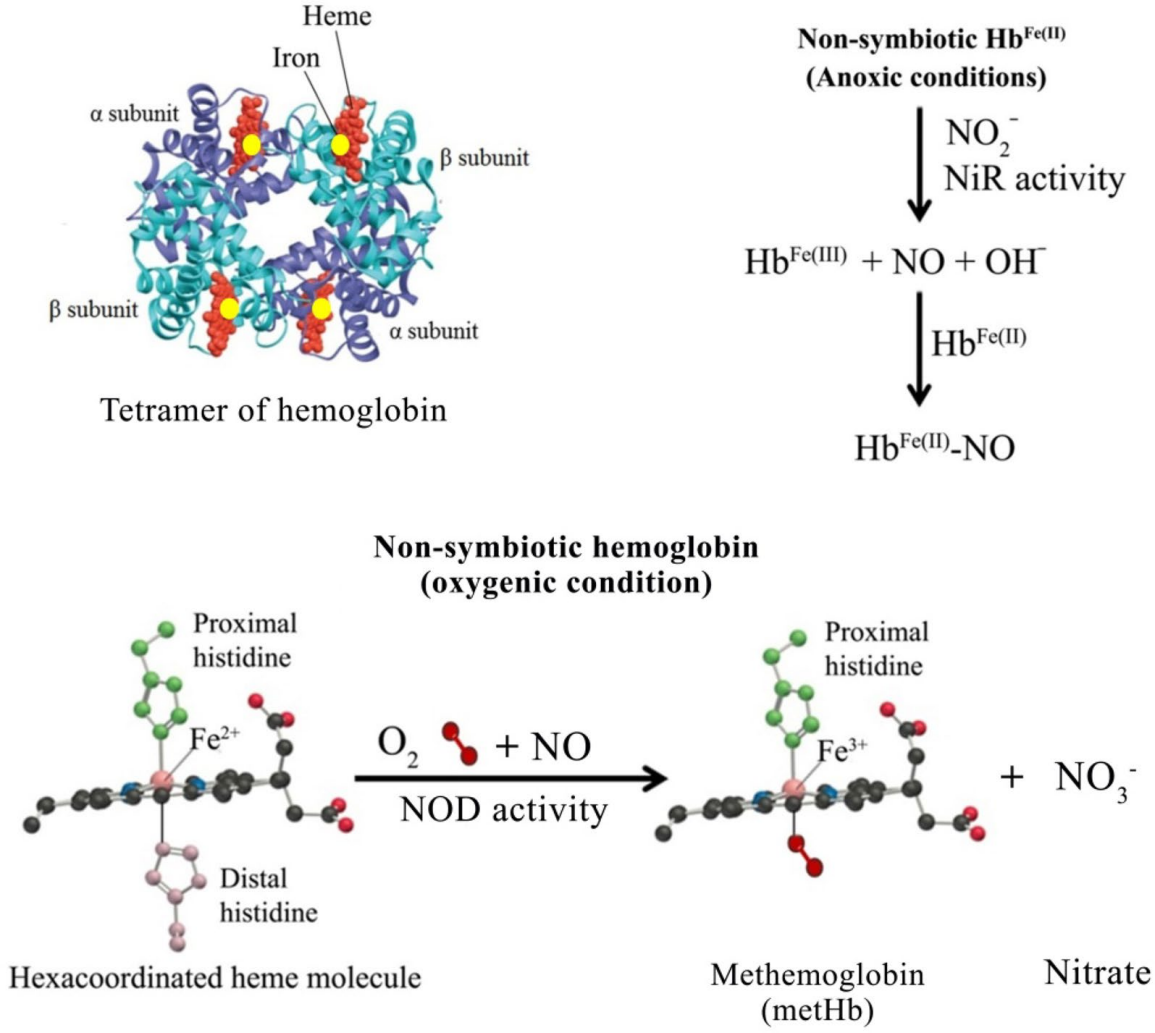
Mechanism of non-symbiotic hemoglobin association with oxygen and NO in anoxic and oxygenic conditions. *NOD* NO-dioxygenase

Hemoglobin is one of the hemoproteins, and NO is considered as a major regulatory component of the function of hemoproteins. NO can either activate or inhibit the activities of various hemoproteins by binding at the metallic center of heme. Furthermore, it is the oxidation state and the coordination environment of the iron center in the hemoproteins which determines the kinetics of NO binding with them [38]. Hexacoordination of heme molecule in non-symbiotic hemoglobin in plants enables it to bind with NO and scavenge it during hypoxic stress conditions [26]. Non-symbiotic hemoglobins possess ligand-binding characteristics different from that of symbiotic hemoglobins. Non-symbiotic Hbs exhibit high rate of oxygen binding than its rate of dissociation compared to symbiotic Hb, which possess high rate of oxygen binding as well as its dissociation. This difference in ligand-binding efficiency of the two proteins is due to differences in heme-coordination state. Thus, in non-symbiotic Hb, heme molecule is hexacoordianted compared to symbiotic Hb where it is pentacoordinated. Hemoglobin binds with both NO and O_2_ depending on the coordination state of heme molecule and performs the functions of either transportation of O_2_ or turnover/scavenging of NO. Symbiotic and erythrocyte hemoglobin is pentacoordinated which allows reversible binding of O_2_. Thus, they are capable of O_2_ transport and storage. However, non-symbiotic Hbs are hexacoordinated and exhibit very high avidity for O_2_. It exist as oxyhemoglobin under most physiological conditions and can efficiently scavenge NO via NO-dioxygenase activity [39–43]. Non-symbiotic hemoglobin expression is affected by a variety of stress conditions, such as hypoxia, cold stress and levels of cellular ATP [26, 27, 31]. In *Arabidopsis*, non-symbiotic Hb (AHb1) exhibits NOD activity in the presence of oxygen (Fig. 4). It removes NO using NADPH as electron donor leading to generation of nitrate and ferric hemoglobin, known as methemoglobin (metHb) [44]. High expression of nsHb in plants exhibits lot of significance as it enables plants to regulate high levels of NO, formed as a result of stress conditions, either by converting NO to nitrate via dioxygenation reaction or by forming nitrosylhemoglobin. Furthermore, it has been suggested that plant lines expressing high levels hemoglobin can prove to be better adapted to both normal and stress conditions [43].

Oxygenated hemoglobin (HbO_2_) is considered as a good choice, and can effectively scavenge NO within concentration range from 125 to 500 µM from both sunflower seedling roots and cotyledons tissue homogenates [21]. The reaction between NO and HbO_2_ is rapid, stoichiometric and leads to formation of methemoglobin and nitrate (NO^3−^) [45]. Due to its size (64.5 kDa), HbO_2_ does not cross cell membranes but can facilitate free diffusion of endogenous NO and its subsequent scavenging in the bathing medium (present work). Furthermore, HbO_2_ works well with the externally applied NO donor which generate NO even in the extracellular compartments, such as NONOates than those which needs intracellular bioactivation to release NO, like organic nitrates. Also, HbO_2_ does not inhibit transnitrosation reactions.

## Conclusion

In view of the above-stated features of hemoglobin and current observations, it is a more reliable alternative to cPTIO as a NO scavenger of tissue NO in pharmacological investigations in plant systems. It (Hb) works efficiently in a concentration-dependent manner in efficiently quenching NO from plant tissues unlike cPTIO, which behaves differently (as NO quencher or as a source of NO) depending on its concentration in the medium. Thus, hemoglobin can be used as an efficient probe for estimation of NO emission from living tissues.

## Abbreviations

NO: nitric oxide
PTIO: 2-phenyl-4,4,5,5,-tetramethylimidazoline-1-oxyl 3-oxide
c-PTIO: carboxy-PTIO
MnIP-Cu: copper derivative of 4-methoxy-2-(1*H*-naphthol (2,3-*d*) imidazol-2-yl) phenol
nsHb: non-symbiotic hemoglobin
metHb: methemoglobin
